# Chloroplast genome sequencing based on genome skimming for identification of Eriobotryae Folium

**DOI:** 10.1186/s12896-021-00728-0

**Published:** 2021-12-11

**Authors:** Fang Li, Xuena Xie, Rong Huang, Enwei Tian, Chan Li, Zhi Chao

**Affiliations:** 1 Department of Pharmacy, Zhujiang Hospital, Southern Medical University, Guangzhou, 510282 China; 2 Faculty of Medicinal Plants and Pharmacognosy, School of Traditional Chinese Medicine, Southern Medical University, Guangzhou, 510515 China; 3 Guangdong Provincial Key Laboratory of Chinese Medicine Pharmaceutics, Guangzhou, 510515 China

**Keywords:** *Eriobotrya japonica*, Eriobotryae Folium, Crude drug, Identification, Chloroplast genome, Genome skimming

## Abstract

**Background:**

Whole chloroplast genome (cpDNA) sequence is becoming widely used in the phylogenetic studies of plant and species identification, but in most cases the cpDNA were acquired from silica gel dried fresh leaves. So far few reports have been available to describe cpDNA acquisition from crude drugs derived from plant materials, the DNA of which usually was seriously damaged during their processing. In this study, we retrieved cpDNA from the commonly used crude drug Eriobotryae Folium (*Pipaye* in Chinese, which is the dried leaves of *Eriobotrya japonica*, PPY) using genome skimming technique.

**Results:**

We successfully recovered cpDNA sequences and rDNA sequences from the crude drug PPY, and bioinformatics analysis showed a high overall consistency between the cpDNA obtained from the crude drugs and fresh samples. In the ML tree, each species formed distinct monophyletic clades based on cpDNA sequence data, while the phylogenetic relationships between *Eriobotrya* species were poorly resolved based on ITS and ITS2.

**Conclusion:**

Our results demonstrate that both cpDNA and ITS/ITS2 are effective for identifying PPY and its counterfeits derived from distantly related species (i.e. *Dillenia turbinata* and *Magnolia grandiflora*), but cpDNA is more effective for distinguishing the counterfeits derived from the close relatives of *Eriobotrya japonica*, suggesting the potential of genome skimming for retrieving cpDNA from crude drugs used in Traditional Chinese Medicine for their identification.

**Supplementary Information:**

The online version contains supplementary material available at 10.1186/s12896-021-00728-0.

## Background

Chloroplast is one of the two organelles having their own genetic materials in plant cells. The chloroplast genomes (cpDNA) are double-stranded DNA in a closed-loop configuration with a length ranging from 120 to 220 kb [1–3]. The cpDNAs, which are maternally inherited and remain haploidy without recombination, have multiple copies per cell and in angiosperms, their size, structure and gene composition are quite consistent [4–7]. The cpDNA contains rich genetic information, based on which a large database can be constructed for comparative study. In addition, the moderate nucleotide substitution rate of cpDNAs and the differences in their molecular evolution speed of the coding region and non-coding region allow for systematic studies of the plants at different levels [8–11]. The good collinearity of the cpDNAs of different plant groups also provides much convenience for comparative analysis and can reflect the phylogenetic history of the plant population [12–15].

The development of high-throughput sequencing technology has allowed full-length sequencing of the cpDNA [16, 17], which has become an important basis of phylogenomic studies. The complete sequence of cpDNA has confirmed some non-genomic-data-based conclusions at different classification levels and revealed many new systematic relationships; it has also shown unique advantages in species identification [12, 13, 18–21]. Using massively parallel sequencing technology, Nock et al. [22] sequenced the cpDNA of *Oryza sativa japonica* and two other *Oryza* species (i.e. *O. meridionalis* and *O. australiensis*), together with that of *Potamophila parviflora* (a close relative to *Oryza*) and *Microlaena stipoides* (an Australian native grass), and found that each species could be identified accurately based on these cpDNA sequences. In the following years, increasing reports emerged on the application of cpDNA sequences in the identification of such medicinal plants as *Magnolia officinalis* [23], *M. grandiflora* [24], *Scutellaria baicalensis* [25], *Fritillaria cirrhosa* [26], and *Ligularia spp.* [27]. According to incomplete statistics, the cpDNA of at least 3721 plant species have been described so far, ranging from green algae to terrestrial and aquatic plants [28].

In almost all these studies, fresh leaves were used as the samples for acquiring cpDNA. No report has been available to describe cpDNA sequencing using samples of crude drugs derived from medicinal plants, the DNA of which was usually damaged during preparation [29, 30]. To investigate the feasibility of cpDNA sequencing based on samples of crude drugs, we attempted to obtain complete chloroplast genome through genome skimming from crude drugs derived from different parts (root, rhizome, fruit and seed) from Pipaye (PPY), the dried leaf of loquat [*Eriobotrya japonica* (Thunb.) Lindl.], the selected representative of leaf-derived crude drugs.

In Traditional Chinese Medicine, PPY is believed to be effective for treating asthma and coughing [31]. Nin Jiom Pei Pa Koa, a Chinese patent medicine with loquat leaf as the main ingredient, has attracted aroused heated discussion in the United States during the influenza season in 2018 after the Wall Street Journal published a report portraying an architect and professor of design at Pratt Institute for taking the medicine to cure his long-standing cough [32]. Actually, the history of using PPY for medical purposes can be dated back to Han Dynasty [33]. In the long history of its medicinal uses, PPY is sometimes confused with the leaves of some other plants, e.g. *Dillenia turbinata* and *Magnolia grandiflora*, which are similar in appearance to loquat leaves [34]. These counterfeits have no effects of genuine PPY, thus should be clearly identified, but their identification can be difficult even for professionals due to their high similarities in appearance, especially when the leaves are cut into pieces.

Theoretically, the Internal transcribed spacer region (ITS) can be used for loquat species identification, but currently no studies of ITS-based identification of PPY against its adulterants has been reported, except for some studies on genetic diversity of *Eriobotrya japonica* [35, 36]; nor was a specific PCR system has been available for PPY identification. Currently, a thin-layer chromatography (TLC) inspection for PPY is recommended in the Chinese Pharmacopoeia, in which ursolic acid serves as the reference substance. As ursolic acid is widely distributed in plant species, the TLC-based identification of crude drugs often has a low specificity. Although a UPLC-Q-TOF/MS analysis targeting the anti-EGFR chemical constituents had been reported for PPY identification [37], the performance of this modality for PPY identification remains to be further verified.

cpDNA sequencing is a promising technique for crude drug identification. Genome skimming is PCR-free to avoid such issues of amplification failure and false positive and false negative results. With genome skimming, not only the cpDNA sequence but also the sequence of ITS region can be obtained from the high-throughput sequencing data, thus a combined analysis of cpDNA and ITS sequences can be possible. Additionally, genome skimming is more cost-effective than MALDI-TOF MS analysis.

In this study, we sequenced the cpDNA not only from fresh leaf samples of *Eriobotrya japonica* and its close relatives *E. deflexa*, *E. cavaleriei*, *E. fragrans*, as well as those of *Dillenia turbinata* and *Magnolia grandiflora*, but also from self-made sun-dried *E. japonica* leaves (self-prepared PPY, SP) and three commercial PPY samples to investigate the feasibility of cpDNA sequencing in identification of the crude drugs. We also compared the efficiency of cpDNA sequencing and the general barcode such as ITS/ITS2 for PPY identification.

## Results

### Analysis of cpDNAs of *Eriobotrya japonica* and its relative and counterfeit species

#### Structure and genes

**Fig. 1:**
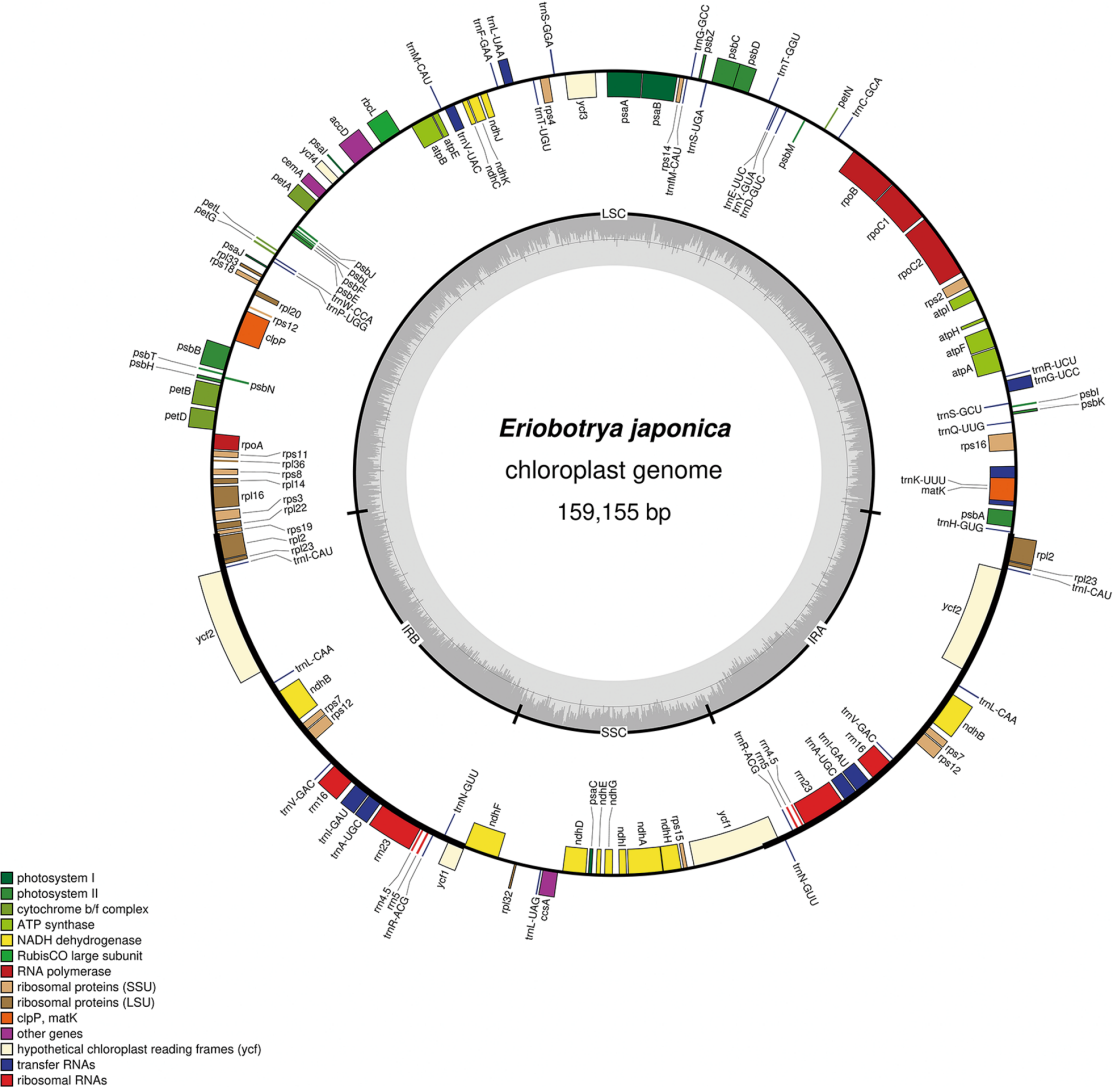
Chloroplast genome map of *E. japonica*. The genes outside of the circle are transcribed clockwise, while those inside are transcribed counterclockwise. Small single copy (SSC), large single copy (LSC), and inverted repeats (IRa, IRb) are indicated

In this study, all the cpDNAs showed a typical circular tetramerous structure, consisting of a pair of inverted repeats (IRs), a large single copy region (LSC), and a small single copy region (SSC) (Fig. 1). The size of cpDNA and its regions were all similar across different *Eriobotrya* species (Table 1). The cpDNA length of genus *Eriobotrya* ranges from 159,115 bp (*E. japonica*) to 159,393 (*E. deflexa*); the cpDNA length is 159,270 bp for *E. cavaleriei* and 159,177 bp for *E. fragrans*. The size of the IR region ranges from 26,317(*E. fragrans*) to 26,335 bp (*E. cavaleriei*), while the SSC and LSC size varies from 19,213 (*E. fragrans*) to 19,350 bp (*E. cavaleriei*) and from 87,222 (*E. japonica*) to 87,401 bp (*E. deflexa*), respectively (Table 1). The cp gonomes of *D. turbinata* and *M. grandiflora* are 163,250–159,690 bp in length, consisting of an IR region of 26,497–26,580 bp, a SSC region of 18,754–19,349 bp and LSC regions of 87,776–90,907 bp. *E. japonica* contains 112 genes, including 78 protein coding genes, 30 tRNA genes and 4 rRNA genes, the same as the remaining *Eriobotrya* species and *M. grandiflora*. The *D. turbinata* cpDNA consists of 113 genes, including 79 protein-coding genes, 30 tRNA genes, and 4 rRNA genes. Compared to the *Eriobotrya* species, *D. turbinata* has 113 genes due to the presence of the gene *infA*. In addition, the presence of *infA* and the deletion of *rpl22* gene of *M. grandiflora* result in the consistency in the number of genes with *Eriobotrya* species. The *ycf1* sequence located in the IRa and SSC boundary of all the samples was identified as a pseudogene because it was truncated, i.e. incomplete duplications of the normal copy. In addition, two pseudogenes, *accD* and *ndhK*, were also found in *D. turbinata*. In the cpDNA of all the samples, the gene *rps12* was a trans-splicing gene, whose 5' exon was located in the LSC region and the 3' exon in the IRs region.

**Table 1:** Summary of cpDNA characteristics of 11 samples

	*E. japonica-*1	*E. japonica-*2	PPY-1	PPY-2	PPY-3	SP	*E. cavaleriei*	*E. deflexa*	*E. fragrans*	*D. turbinata*	*M. grandiflora*
DNA concentration (ng/μL)	273.1	310.1	41.9	29.4	33.6	9.76	88	21.9	47.5	43.8	362.7
Coverage	309.71 ± 45.78	747.45 ± 99.19	60.02 ± 20.91	140.14 ± 28.56	188.80 ± 35.90	59.99 ± 20.96	505.66 ± 44.15	579.12 ± 94.80	492.26 ± 54.56	359.61 ± 71.08	258.53 ± 36.78
Size (bp)	159,115	159,156	159,155	159,156	159,156	159,202	159,270	159,393	159,177	163,250	159,690
Number of genes	112	112	112	112	112	112	112	112	112	113	112
Number of protien-coding genes	78	78	78	78	78	78	78	78	78	79	78
Number of tRNA genes	30	30	30	30	30	30	30	30	30	30	30
Number of rRNA genes	4	4	4	4	4	4	4	4	4	4	4
Overall GC content	36.70%	36.70%	36.70%	36.70%	36.70%	36.70%	36.70%	36.70%	31.00%	36.10%	39.3%
LSC length (bp)	87,222	87,223	87,222	87,223	87,223	87,271	87,250	87,401	87,330	90,907	87,776
IR length (bp)	26,326	26,326	26,326	26,326	26,326	26,325	26,335	26,330	26,317	26,497	26,580
SSC length (bp)	19,281	19,281	19,281	19,281	19,281	19,281	19,350	19,332	19,213	19,349	18,754
GC content in IR (%)	42.70%	42.70%	42.70%	42.70%	42.70%	42.70%	42.70%	42.70%	42.70%	42.80%	42.70%
Reference sequence	KY085905, MN577877, NC034639 for cpDNA MH711704, MG938044, MF096288, KX675082 for ITS/ITS2						MN577877 for cpDNA KJ170784, KP093136 for ITS/ITS2	MK920282 for cpDNA MG938042, JQ392434 for ITS/ITS2	MN577877 for cpDNA MH246945, KP093137 for ITS/ITS2	NC042740 for cpDNA AY096031 for ITS/ITS2	JN867587, NC020318, MN990594 for cpDNA

**Fig. 2:**
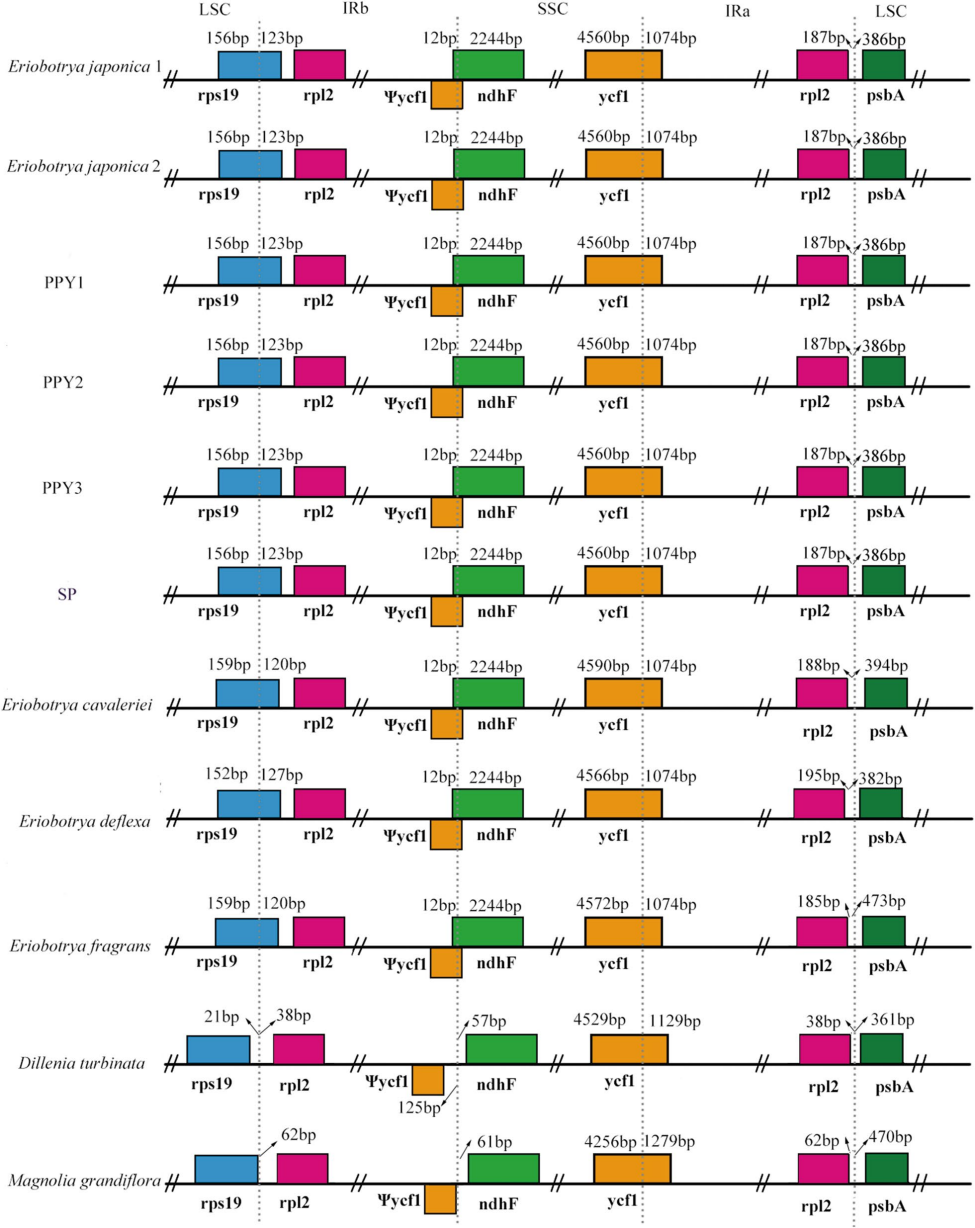
Comparison of the border regions of the LSC, SSC and IR regions among the 11 cp genomes. The genes cross the LSC/IRb or IRb/SSC regions, indicating that the LSC/IRb boundary has moved backward or the IRb/SSC boundary moves forward in these species

The junction positions were conserved in *Eriobotrya* species. *Eriobotrya* species have partially duplicated *rps19* and *ndhF* genes in the IR regions, while these two genes are located respectively in the LSC and SSC regions of *D. turbinata* and *M. grandiflora* (Fig. 2). In *Eriobotrya* species, the extent of *rpsl9* duplication ranges from 120 (*E. cavaleriei* and *E. fragrans*) to 127 bp (*E. deflexa*), and 12 nucleotides of *ndhF* are duplicated. The final 12 nucleotides of the IR region are shared by *ndhF* and the pseudogene *ycf1* (*ψycf1*), which are transcribed in opposite directions; in *D. turbinata* and *M. grandiflora*, *ψycf1* gene is located in the IRb region and *ndhF* in the SSC region. The LSC/IRa-*rpl2* spacer ranges in length between 38 (*D. turbinata*) and 195 nucleotides (*E. deflexa*).

#### Condon usage

**Fig. 3:**
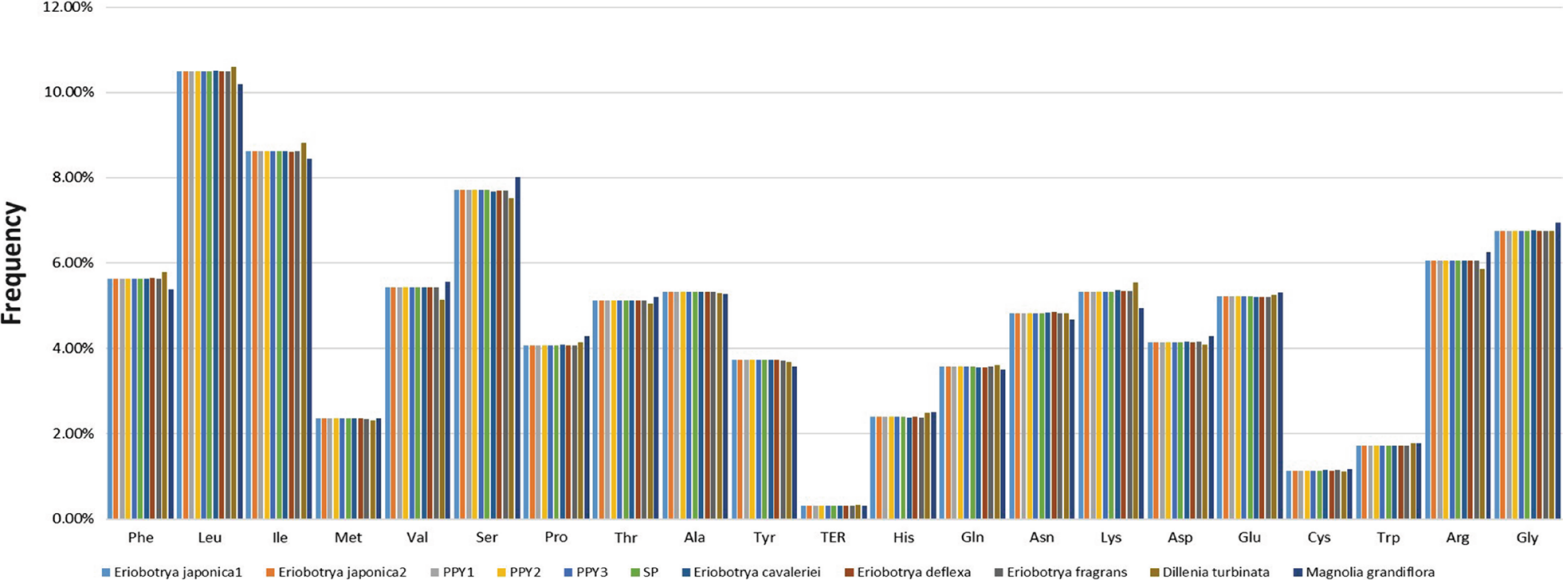
Amino acid frequencies in 11 samples protein-coding sequences

The total length of the protein coding genes (PCGs) of *Eriobotrya* cpDNAs ranges from 78,600 (*E. fragrans*) to 78,630 bp (*E. cavaleriei* and *E. deflexa*), and that of *D. turbinata* and *M. grandiflora* was 77,301 bp and 77,811 bp, respectively (Table 2). These PCGs contain 25,767 (in *D. turbinata*) to 26,210 (in *E. cavaleriei* and *E. deflexa*) codons, with UGA, UAG and UAA as the termination codons. For *Eriobotrya* cpDNAs, the most frequent amino acid is leucine (Leu), encoded by 2749–2754 (10.51%) of the codons; the least frequent amino acid in the cpDNAs is cysteine (Cys), encoded by 299–301 (1.14%) of the codons (Fig. 3). Most of the amino acid codons have preferences except for methionine and tryptophan. Within the PCGs of *Eriobotrya* cpDNAs, the GC content of the codons in the third position was 26.7%. Within the PCGs of *D. turbinata* and *M. grandiflora* cpDNAs, the AT content of the codons at the third position is 26.4% and 28.8%, respectively. All the preferred synonymous codons (RSCU > 1) of *E. japonica* ended with A or U except for the codons of *trnL-CAA*, while most of the non-preferred synonymous codons (RSCU < 1) ended with G or C, which is the same as the other *Eriobotrya* species in our study.

**Table 2:** Indexes of codon usage bias in 11 samples representing 6 species

	*E. japonica*-1	*E. japonica*-2	PPY-1	PPY-2	PPY-3	SP	*E. cavaleriei*	*E. deflexa*	*E. fragrans*	*D. turbinata*	*M. grandiflora*
PCG length (bp)	78,612	78,612	78,612	78,612	78,612	78,612	78,630	78,630	78,600	77,301	77,811
Codon Number	26,204	26,204	26,204	26,204	26,204	26,204	26,210	26,210	26,200	25,767	26,122
Amino acid No	26,120	26,120	26,120	26,120	26,120	26,120	26,126	26,126	26,116	25,682	26,038
SC No	25,052	25,052	25,052	25,052	25,052	25,052	25,058	25,058	25,049	24,632	24,959
ENC	49.52	49.52	49.52	49.52	49.52	49.52	49.51	49.52	49.52	49.33	50.66
CAI	0.166	0.166	0.166	0.166	0.166	0.166	0.166	0.166	0.165	0.165	0.167
CBI	− 0.106	− 0.106	− 0.106	− 0.106	− 0.106	− 0.106	− 0.106	− 0.106	− 0.107	− 0.114	− 0.096
FOP	0.350	0.350	0.350	0.350	0.350	0.350	0.350	0.350	0.350	0.346	0.357
GC content (%)	0.378	0.378	0.378	0.378	0.378	0.378	0.378	0.378	0.378	0.375	0.392
GC_3_ content (%)	0.267	0.267	0.267	0.267	0.267	0.267	0.267	0.267	0.267	0.264	0.288

#### SSRs and long repeat sequences

We found that the mononucleotide repeats of genus *Eriobotrya*, *D. turbinata* and *M. grandiflora* were by far the most frequent SSR type, followed by dinucleotides, tetranucleotides, trinucleotides, pentanucleotides, and finally hexanucleotide (Table 3). *Eriobotrya* cpDNAs exhibit variations in the number of SSRs; the number is 92 in *E. japonica*, 90 in *E. cavaleriei*, 108 in *E. deflexa* and 98 in *E. fragrans*. The number of SSRs is 93 in *D. turbinata*, and is only 53 in *M. grandiflora*, the smallest among all the species. among the *Eriobotrya* species, there was no trinucleotide repeat and only a single hexanucleotides was found only in *E. deflexa*. No pentanucleotide repeat was found in *M. grandiflora*.

**Table 3:** Comparison of simple repeats (SSR) in 11 cp genomes

Sample	Mononucleotides	Dinucleotides	Trinucleotides	Tetranucleotides	Pentanucleotides	Hexanucleotides	Total
*E. japonica*-1	70	15	0	6	1	0	92
*E. japonica*-2	70	15	0	6	1	0	92
PPY-1	70	15	0	6	1	0	92
PPY-2	70	15	0	6	1	0	92
PPY-3	70	15	0	6	1	0	92
SP	70	15	0	6	1	0	92
*E. cavaleriei*	70	15	0	4	1	0	90
*E. deflexa*	83	17	0	6	1	1	108
*E. fragrans*	75	17	0	6	0	0	98
*D. turbinata*	58	18	6	9	1	1	93
*M. grandiflora*	30	9	3	9	0	2	53
Total	736	166	9	70	9	4	994
Ratio	74.04%	16.70%	0.91%	7.04%	0.91%	0.40%	100%

The tandem repeats in the cpDNAs of *Eriobotrya* species has generally a low variation, ranging from 130 (*E. fragrans*) to 133 (*E. cavaleriei*) (Table 4). Among all the species, *D. turbinata* has the highest number of tandem repeats (up to 216), while *M. grandiflora* has the least number of only 49. Five different long repeats, including tandem, complement, forward, palindromic and reverse repeats, were found in the cpDNA in this study. Complement repeat was absent in *E. japonica, E. fragrans* and *M. grandiflora.* Reverse repeat was not found in *M. grandiflora*.

**Table 4:** Comparison of long repeat sequences in 11 cp genomes

Sample	Tandem repeat	Complement repeat	Forward repeat	Palindromic repeat	Reverse repeat	Total
*E. japonica*-1	131	0	25	20	3	179
*japonica-2*	131	0	25	20	3	179
PPY-1	131	0	25	20	3	179
PPY-2	131	0	25	20	3	179
PPY-3	131	0	25	20	3	179
SP	133	0	26	20	3	182
*E. cavaleriei*	133	2	23	18	7	183
*E. deflexa*	132	1	22	16	11	182
*E. fragrans*	130	0	22	17	11	180
*D. turbinata*	216	1	19	19	8	263
*M. grandiflora*	49	0	11	16	0	76
total	1448	4	248	206	55	1961

#### Highly divergent regions

**Fig. 4:**
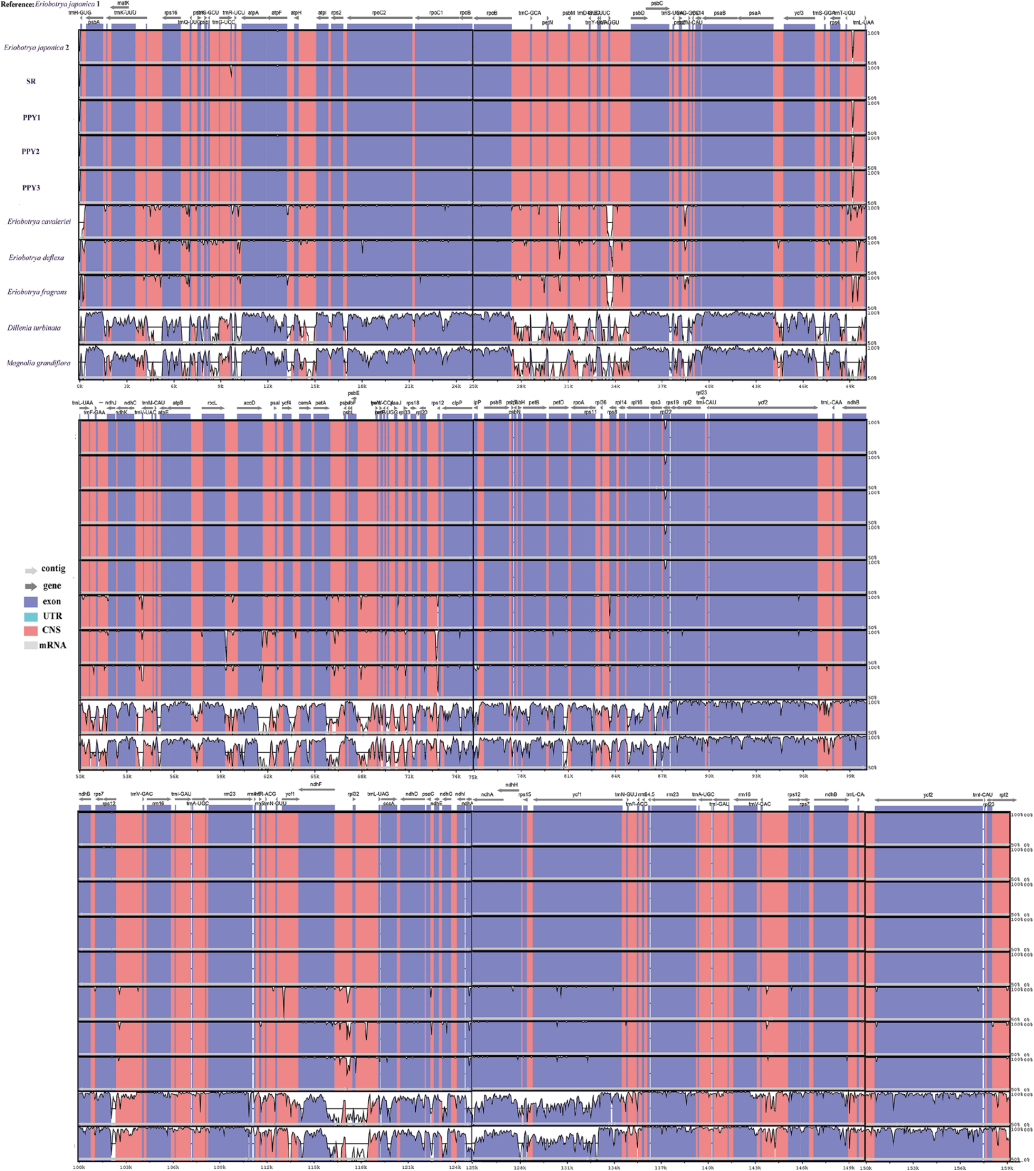
Comparative chloroplast genomic analysis. The red area represents the non-coding area, and the purple area represents the coding area. The large twists and turns indicate large variations

**Fig. 5:**
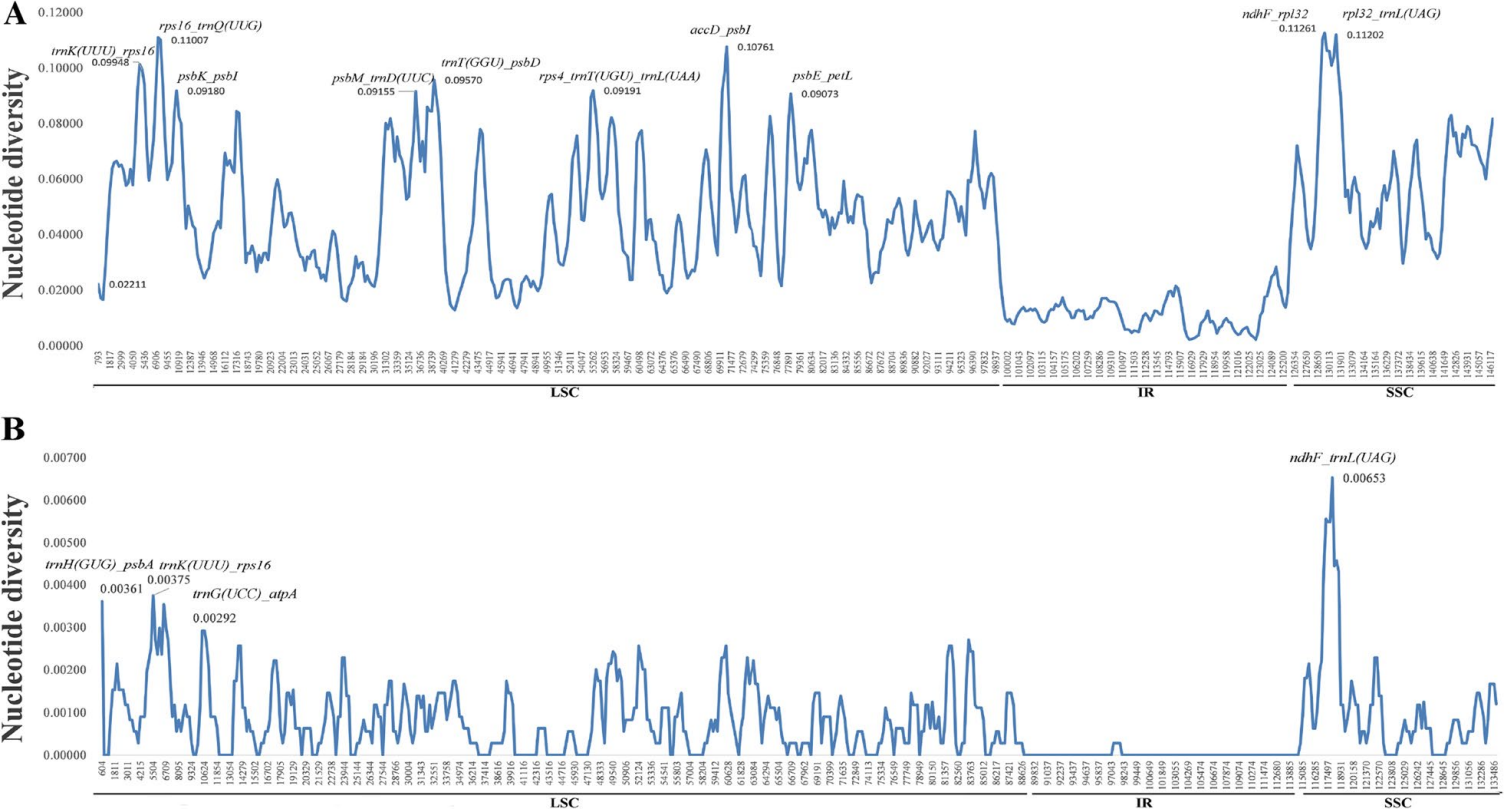
Comparative analysis of the nucleotide diversity (Pi) value of the cp genomes among the 11 species. **A** Coding regions, **B** non-coding regions

In the cpDNA of each species, the non-coding regions have a greater variability than the coding regions (Fig. 4). Several divergent regions such as *trnH-GUG*, *petN-psbM*, and *trnT-GGU-psbD* were found in *Eriobotrya* species. For all the species, some highly variable regions were observed in the intergenic regions, as in *trnH-GUG*, *trnK-UUU-rps16*, *petN-psbM*, *trnT-GGU-trnL-UAA*, *rpl20-rps12, psbZ-trnG-GCC* (Fig. 5). The *ndhF-rpl32* region showed the highest average sequence divergence (0.1126), followed by *rpl32-trnL-UAG* (0.1202), *rps16-trnQ-UUG* (0.11007), and *accD-psbI* (0.1076) (Fig. 5), with the remaining genes having a divergence less than 0.1.

### Comparison of cpDNAs obtained from PPY, SP and *E. japonica* fresh leaves

The average cover of fresh samples of *E. japonica* (309.71–747.45) was as high as about 5 times that of the dried samples (59.99–188.80). Both PPY and SP were consistent with *E. japonica* in terms of gene number, GC content (Table 1), genetic makeup (Table 5), the boundaries of IR region (Fig. 2), codon usage (Table 2), and SSRs type and number (Table 3). Both of PPY and SP had 112 genes with a GC content of 36.7%, including 78 protein coding genes, 30 tRNA genes and 4 rRNA genes. In structural analysis of cpDNAs, only minor variations were observed in terms of the length of cpDNAs (from 159,115 bp in *E. japonica* to 159,202 bp in SP) (Table 1) and the amount of long repeat sequences (Table 4). SP had one more forward repeats and two more tandem repeats than *E. japonica,* while PPY was similar with *E. japonica* in the amount and type of the long repeat sequences.

**Table 5:** List of genes found in *Eriobotrya japonica* cpDNA

Gene category	Gene group	Gene name
Photosynthesis related genes	Photosystem I	*psaA, psaB, psaC, psaI, psaJ*
	photosystem II	*psbA, psbB, psbC, psbD, psbE, psbF, psbH, psbI, psbJ, psbK, psbL, psbM, psbN, psbT, psbZ*
	Cytochrome b/f complex	*petA*(× 2), petB*, petD*, petG, petL, petN
	ATP synthase	*atpA, atpB, atpE, atpF, atpH, atpI*
	NADH dehydrogenase	ndhA, ndhB*(× 2), *ndhC, ndhD, ndhE, ndhF, ndhG, ndhH, ndhI, ndhJ, ndhK*
	RubisCO large subunit	*rbcl*
Transcription and translation related genes	Ribosomal proteins(SSU)	*rps2, rps3, rps4, rps7*(× 2), rps8, rps11, rps12**(× 2), rps14, rps15, rps16*, rps18, rps19
	Ribosomal proteins(LSU)	rpl2*(× 2), rpl14, rpl16*, rpl20, rpl22, rpl23(× 2), *rpl32, rpl33, rpl36*
RNA genes	Ribosomal RNAs	*rrn4.5*(× 2)*, rrn5*(× 2)*, rrn16*(× 2)*, rrn23*(× 2)
	Transfer RNAs	*trnS-GGA, trnS-UGA, trnS-GCU, trnE-UUC, trnT-UGU, trnT-GGU, trnF-GAA, trnM-CAU, trnW-CCA, trnP-UGG, trnI-CAU*(× 2), trnI-GAU*(× 2)*, trnL-CAA*(× 2), trnL-UAA*, trnL-UAG, trnV-GAC(× 2), trnV-UAC*, trnR-ACG(× 2)*, trnR-UCU, trnN-GUU*(× 2), trnH-GUG, trnQ-UUG, trnC-GCA, trnD-GUC, trnY-GUA, trnG-UCC*, trnfM-CAU, trnK-UUU*, trnA-UGC*(× 2), *trnG-GCC*
	RNA polymerase	ropA, ropB, ropC1*, ropC2
Other genes		ccsA, accD, cemA, clpP**, matK
Proteins of unknown function	ycf	*ycf1, ycf2*(× 2), ycf3**, ycf4

### Phylogenetic tree and species identification

**Fig. 6:**
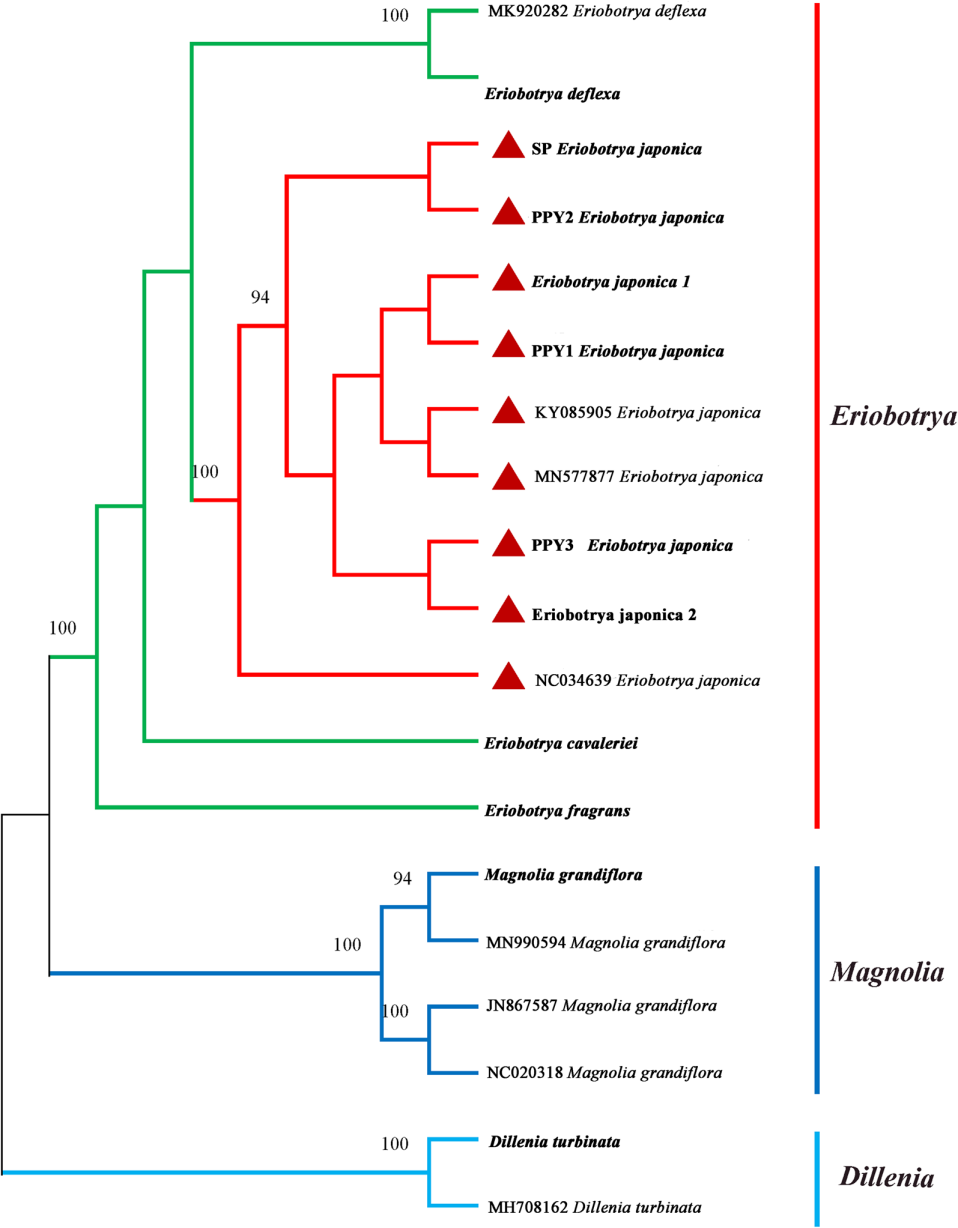
Phylogenetic tree constructed using ML based on complete cp genomes. The number above the branches are bootstrap support values

**Fig. 7:**
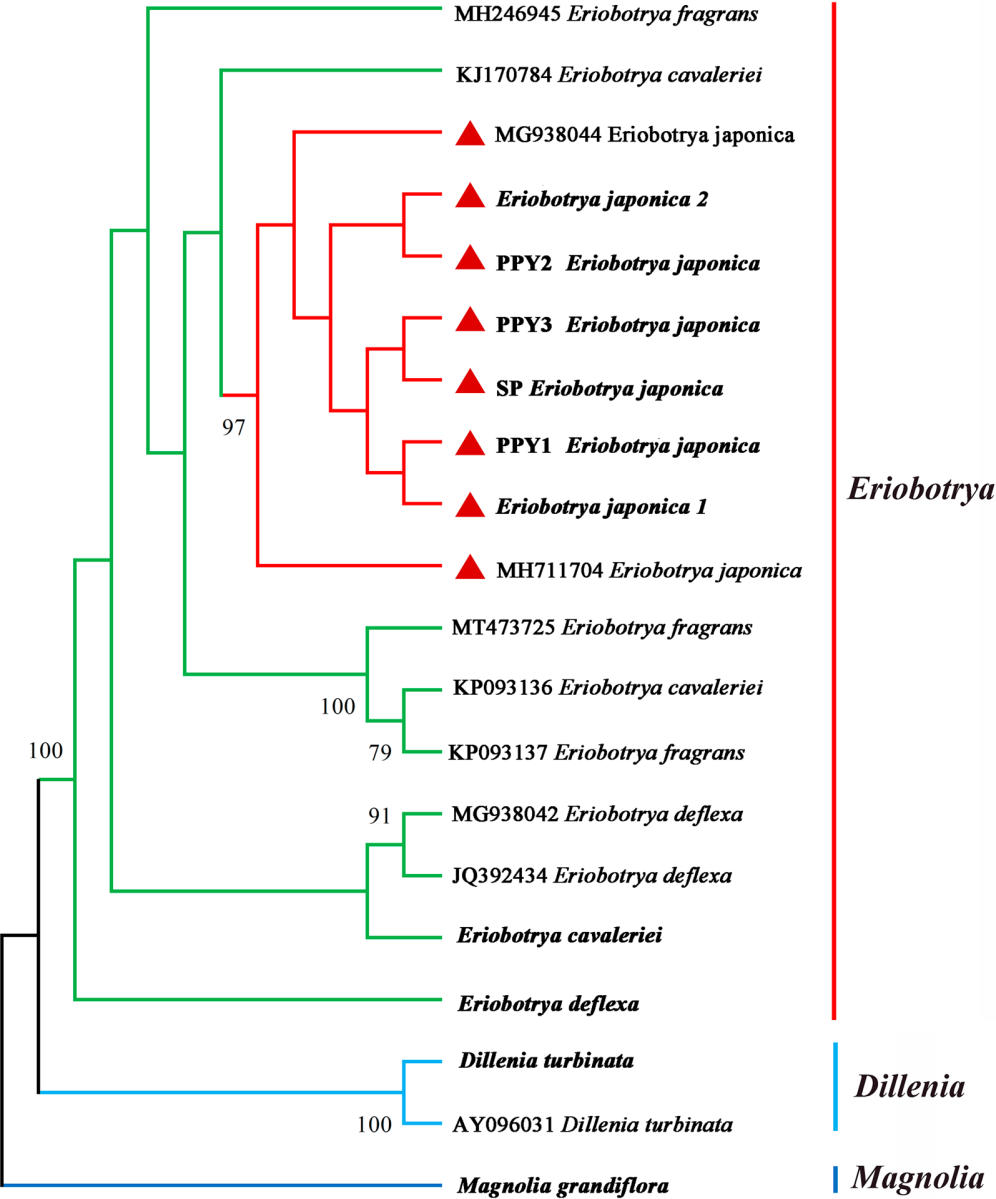
Phylogenetic tree constructed using ML tree based on 20 ITS sequences. The numbers above the branches are bootstrap support values

Among all the species, the topological structures of ITS, ITS2 and cpDNAs were basically identical, including three major clades, namely *Eriobotrya*, *Dillenia* and *Magnolia* species (Figs. 6, 7, Additional file 1: Fig. S1). But the phylogenetic positions based on ITS and ITS2 of the other *Eriobotrya* species were different in that *E. cavaleriei* was placed close to *E. deflexa* or *E. fragrans* with strong support (Fig. 7; Additional file 1: Fig. S1). In addition, the *Dillenia* species was closely related to *Eriobotrya* species, as shown in Fig. 7. The ML tree based on cpDNA had a higher resolution and each genus node had a bootstrap value of 100% (Fig. 6). PPY, SP and *E. japonica* were all classified into one clade with a bootstrap value of 100%.

Based on the K2P model, the intraspecific genetic distances ranged from 0.0005 (*E. japonica*) to 0.0889 (*E. cavaleriei*), from 0.0026 (*E. japonica*) to 0.1403 (*E. fragrans*), and from 0.0000 (*E. japonica*) to 0.0004 (*M. grandiflora*) in the cases of ITS, ITS2, and cpDNA, respectively; the interspecific genetic distances ranged from 0.0285 (*E. japonica* and *E. deflexa*) to 0.8665 (*M. grandiflora* and *D. turbinata*), from 0.0371 (*E. japonica* and *E. deflexa*) to 0.7495 (*M. grandiflora* and *E. fragrans*), and from 0.0007 (*E. japonica* and *E. deflexa*) to 0.1195 ((*M. grandiflora* and *D.indica*), respectively.

## Discussion

The cpDNA of higher plants is highly conserved, which ensures the direct homology of genes among distant evolutionary groups. Compared with nuclear and mitochondrion genome, cpDNA has a greater gene density with a moderate evolution rate, thus making cpDNA a suitable and unique molecule for accurate species identification. Currently few studies have been available to report plant species identification by sequencing cpDNA from crude drugs derived from plants instead of fresh leaves. To test the feasibility of acquiring complete cpDNA through genome skimming for crude drug identification, we used commercial PPY samples purchased from local pharmacies, i.e. the crude drug practically sold to patients, not merely silica gel dried fresh leaf materials used in previous studies. To our best knowledge, such a pilot empirical study has not been reported previously.

Different from that in silica gel dried fresh leaf materials, the genomic DNA in crude drugs usually have severe degradation, as often seen in the specimens stored for a long time. Long storage time can result in DNA degradation [30] and DNA fragmentation [29] to cause difficulties in the genome sequencing and identification. Genome skimming has proved to well suit the needs of species identification based on degenerated genome DNA, and researchers have successfully sequenced cpDNA from herbarium materials stored for decades with this technique [38–40], which is even capable of sequencing complete or almost complete cpDNA from specimens stored up to 146 years.

As expected, the genomic DNA extracted from the crude drugs was of a poor quality in this study. But with genome skimming, the cpDNAs retrieved were almost identical to those obtained from the fresh samples, and a low amount of degraded genomic DNA (9 ng) was sufficient for operation. cpDNAs acquired from PPY, SP and *E. japonica* samples showed negligible variations, which can be inferred from the same coding genes, tRNAs and rRNAs among their cp genomes. Besides cpDNA, we also successfully recovered rDNA sequences from the crude drug PPY. These results further demonstrate that genome skimming is less affected by template quality than other sequencing methods [38–41].

In the continuous efforts for searching ideal DNA barcodes for plants, ITS/ITS2 have been considered as the most promising ones [42, 43] for their high resolution of inter- and intraspecific relationship [44–47], but so far a widely accepted universal DNA barcode has not been available yet. Appropriate barcodes for specific plant taxonomic groups should be investigated case by case. Theoretically, ITS/ITS2 can be used for *Eriobotrya* species identification with better convenience and at a lower cost compared to cp genome method. Nevertheless, our results confirmed that both cpDNA and ITS/ITS2 were efficient for identifying PPY and its simple counterfeits (*Dillenia turbinata* and *Magnolia grandiflora*), but ITS/ITS2-based identification had a poor resolution for *Eriobotrya* species, *E. japonica* and its close relatives (*E. deflexa, E. cavaleriei, E. fragrans*). Previous studies proposed that the unresolved relationships among them may be attributed to the confusion of the interspecific boundaries between *E. cavaleriei* and *E. fragrans* based on short sequences [48–50]. Overlaps between the intraspecific and interspecific K2P distances based on ITS/ITS2 were also reported. Thus, the short sequences (i.e. rDNA ITS/ITS2) are not as powerful as expected in identifying Eriobotryae Folium and its counterfeits due to insufficient variation information.

CpDNA contains much more genetic information and can provide a large database for species identification [12, 51–53] to significantly increase the resolution at lower taxonomic ranks such as genus and species, and thus may serve as a super barcode for species identification [26], as in the case of *Eriobotrya*. Our phylogenetic analysis based on cpDNA data showed that the samples belonging to the same species formed a separate clade, each with a high bootstrap value. In addition, the intraspecific K2P distance values were significantly lower than the interspecific K2P distance when using cpDNA data. These results demonstrate that, compared to ITS and ITS2 sequences, cpDNA is more effective for the identification of Eriobotryae Folium.

Although cpDNA genome can provide more characteristics and increase the amount of sequence data to enhance species discrimination, it does not address the basic challenge that cpDNA do not necessarily track species boundaries [54]. Substantial numbers of unlinked nuclear markers (e. g. transcriptome sequencing and RAD-seq) should be taken to access the ultimate big gains in the discriminatory power [54].

## Conclusions

Despite of severe degradation of the genomic DNA, cpDNA and rDNA can be successfully sequenced and assembled from crude drug of Eriobotryae Folium through genome skimming. Chloroplast genome sequence data can be more effective than rDNA ITS and ITS2 sequences for the identification of Eriobotryae Folium and the counterfeits with a close resemblance. The results of this study demonstrate that genome skimming is capable of retrieving whole chloroplast genome from crude drugs used in traditional Chinese medicine for their identification.

## Methods

### Plant and crude drug samples

Two samples of fresh leaves of *E. japonica* (*E. japonica*-1 and *E. japonica*-2) were collected from the Medicinal Plant Garden of Southern Medical University and South China Botanical Garden. The fresh leaves of *E. cavaleriei*, *E. deflexa*, *E. fragrans*, *D. turbinata* and *M. grandiflora* were collected from different localities. A portion of the sample *E. japonica*-1 was subjected to sun-drying to prepare self-made PPY sample (SP). Three batches of PPY crude drug (PPY-1, PPY-2 and PPY-3) were purchased from Kangmei Pharmaceutical Co., Ltd, Dongfang Pharmacy, and Henglu Pharmacy, respectively. The voucher specimens and crude drug samples were all identified by the corresponding author (Table 6). The crude drug samples were kept in a cool and dry place, while the fresh leaf samples were kept at − 80 °C.

**Table 6:** Information of samples

Samples	Collecting site locality	Geographical coordinates	Specimen voucher/batch no.	GenBank accession of cp genome
*Eriobotrya japonica-*1	Medicinal Plant Garden of Southern Medical University	23° 19′ 45″ N, 113° 34′ 37″ E	Chao Zhi EJ201403	MT479167
*E. japonica-*2	South China Botanical Garden	23° 19′ 23″ N, 113° 37′ 18″ E	Chao Zhi EJ201910	MT473726
*E. cavaleriei*	Wuhan Botanical Garden	30° 54′ 49″ N, 114° 43′ 30″ E	Chao Zhi 201,812	MT473722
*E. deflexa*	Guangdong Tree Park	23° 20′ 13″ N, 113° 38′ 05″ E	Chao Zhi ED201812	MT473724
*E. fragrans*	Chenhedong Nature Reserve, Guangdong	23° 44′ 02″ N, 113° 50′ 64″ E	Chao Zhi EF201903	MT473725
*Dillenia turbinata*	South China Botanical Garden	23° 18′ 51″ N, 113° 36′ 77″ E	Chao Zhi DT201403	MT473723
*Magnolia grandiflora*	Medicinal Plant Garden of Southern Medical University	23° 19′ 45″ N, 113° 34′ 37″ E	Chao Zhi MG201403	MT473732
SP	prepared from *E. japonica*-1	–	–	MT473731
PPY-1	Kangmei Pharmaceutical Co., Ltd, Guangdong	–	YC20181201	MT473727
PPY-2	Dongfang Pharmacy, Guangzhou	–	YC20181202	MT473728
PPY-3	Henglu Pharmacy, Guangzhou	–	YC20181203	MT473730

### DNA extraction

Genomic DNA was extracted from the above samples using the modified CTAB method [55]. To eliminate the interference by phenolic substances on DNA extraction, 20 mg polyvinyl pyrrolidone was mixed with *Eriobotrya* samples before DNA extraction [56]. DNA concentration and quality were examined using a NanoDrop 2000C spectrophotometer and by 1.2% agarose gel electrophoresis.

### Sequencing, genome assembly and annotation

Approximately 1 μg genomic DNA was randomly fragmented by Covaris (E210), followed by fragments selection by Agencourt AMPure XP-Medium kit to an average size of 200–400 bp. Selected fragments were end-repaired and 3’adenylated, and the resulting DNA was ligated with adaptors. After the ligation, the products were amplified by PCR and purified using Agencourt AMPure XP-Medium kit. The purified double-stranded PCR products were heat-denatured to single stand and circularized by the splint oligo sequence to generate a single strand circular DNA (ssCir DNA) library after quality control. The ssCir DNA molecule formed a DNA nanoball (DNB), and the final DNB was loaded onto a sequencing chip and were sequenced using the BGISEQ-500 platform. Finally, the pair-end (PE) 124–150 bp reads were obtained by combinatorial Probe-Anchor Synthesis (cPAS).

Low-quality reads, adapter contamination, and duplicated reads were removed from the PE sequence data generated from the BGI platform using SOAPnuke software v2.1.5 [57] to produce the “clean data”, which were filtered using Bowtie2 [58] and then assembled using SPAdes v3.14.0 [59] in GetOrganelle v1.7.0 [60]. In cases of failure of ribosomal DNA assembly, we amplified and sequenced the ribosomal DNA to obtain the ITS and ITS2 sequences. To improve genome assembly, we also conducted reference-based genome assembly using the cpDNA sequences available in GenBank (Table 1). The contigs obtained from the GetOrganelle assemblies were aligned to the reference genome, and the aligned contigs were assembled to each cpDNA in Geneious v2020.0.4 [61].

The assembled cpDNAs were annotated using GeSeq (Annotation of Organellar Genomes) (https://chlorobox.mpimp-golm.mpg.de/geseq.html) [62] and Plastid Genome Annotator (PGA) [63] software, followed by manual adjustments of the start and stop codons and the exon and intron boundaries via Geneious. The ribosomal DNA was annotated using Geneious. All the tRNA genes were confirmed using the online tRNAscan-SE v2.0.7 [64, 65] and ARAGORN v1.2.38 [66]. The OGDRAM (http://ogdraw.mpimp-golm.mpg.de/) [67] software was used to draw the circular cpDNA maps. The annotated cpDNAs and the ribosomal DNA sequence were submitted to GenBank (http://www.ncbi.nlm.nih.gov/) to obtain the accession number (Table 2). The IR and SSC boundary regions of *E. japonica* species were compared and examined with other cpDNAs.

### Genome structure and comparative analysis

CpDNA characteristics (e. g. structure and genes; codon usage, SSRs and long repeat sequences) were compared among the species concerned for species identification. To determine whether the chloroplast genome sequences of PPY and SP obtained herein were complete, we also compared cpDNA characteristics between PPY/SP and fresh samples. The codon usage and the relative synonymous codon usage values (RSCU) of cpDNAs exons in the consensus protein-coding genes of each species were obtained using CondoW v1.4.2 [68]. The MISA software v2.1 [69] was used to predict the simple repeats (SSR) in cpDNA using the following parameter setting: mononucleotide repeat number > 10, dinucleotide repeat number > 5, trinucleotide repeat number > 4, tetranucleotide, pentanucleotide and hexanucleotide repeat number > 3; the minimum distance between two SSRs was set as 100 bp. If the distance between two SSRs was less than 100 bp, the two SSRs were regarded as one composite microsatellite. The Tandem Repeats Finder was used to predict the tandem repeats with parameters of 2 for the matching weight, 5 for the penalty on the mismatching and the indel, the minimum alignment score to report repeat was set to 50, and 500 for the maximum period size to report [70]. Repeat sequences were predicted by the website REPuter [71]. The minimum repeat size was set to 30 bp, and the sequence identity with Hamming distance was 3. The cpDNA of *E. japonica* was used as the reference sequence, and the sequence similarity of cpDNA was analyzed by Shuffle-LAGAN mode of mVISTA [72].

### Phylogenetic analysis and tree-based identification

The identification capability of cpDNA and the universal barcode regions were compared by constructing a maximum likelihood (ML) tree based on complete cpDNA, ITS and ITS2. Additional nine ITS sequences, two ITS2 sequences and eight cpDNA sequences were also downloaded from GenBank (Additional file 1: Table S4) to enrich the data set. The cpDNAs, ITS, and ITS2 sequences of all species in this study and the published genomes from GenBank were aligned using MAFFT v7.037 [73] and adjusted manually with MEGA6 software as needed [74]. The cpDNA sequences downloaded from GenBank were listed in Table 1. The best-fit substitution models for these cpDNA sequences were inferred by ModelFinder [75] integrated into PhyloSuite [76] based on the Akaike Information Criterion (AIC). Phylogenetic trees were constructed by ML using RAxML (v8.2.4) with the GTR + F + G4 model [75] and 1000 bootstrap replicates. The genetic distance between the species in this study and the reference sequences mentioned above was calculated based on the Kimura 2-parameter distance (K2P) model [77].

## Supplementary Information

**Additional file 1: Fig. S1.** Phylogenetic tree constructed using ML tree based on 20 ITS2 sequences. The number above the branches are bootstrap support values. **Table S1.** Interspecific (below diagonal) and intraspecific (diagonal) genetic distance of cp genomes of six species. **Table S2.** Interspecific (below diagonal) and intraspecific (diagonal) genetic distance of ITS of six species. **Table S3.** Interspecific (below diagonal) and intraspecific (diagonal) genetic distance of ITS2 of six species. **Table S4.** Additional ITS/ITS2 and cpDNA sequences downloaded from the GenBank to construct ML tree.

## Data Availability

The complete chloroplast genomes of 11 samples were submitted to the NCBI database (https://www.ncbi.nlm.nih.gov/). All other data and material generated in this manuscript are available from the corresponding author upon reasonable request.

## Abbreviations

cpDNA: Chloroplast DNA
ITS: Internal transcribed spacer
IR: Inverted repeat
LSC: Large single-copy
ML: Maximum likelihood
SSC: Small single-copy
