# Supplementary material for: Chloroplast genome sequencing based on genome skimming for identification of Eriobotryae Folium
Source: BMC Biotechnol. 2021 Dec 11;21:69. doi: 10.1186/s12896-021-00728-0 (PMC8666020; doi:10.1186/s12896-021-00728-0)
Supplement: Supplementary file 1 — Additional file 1: Fig. S1. Phylogenetic tree constructed using ML tree based on 20 ITS2 sequences. The number above the branches are bootstrap support values. Table S1. Interspecific (below diagonal) and intraspecific (diagonal) genetic distance of cp genomes of six species. Table S2. Interspecific (below diagonal) and intraspecific (diagonal) genetic distance of ITS of six species. Table S3. Interspecific (below diagonal) and intraspecific (diagonal) genetic distance of ITS2 of six species. Table S4. Additional ITS/ITS2 and cpDNA sequences downloaded from the GenBank to construct ML tree. [file 12896_2021_728_MOESM1_ESM.docx]

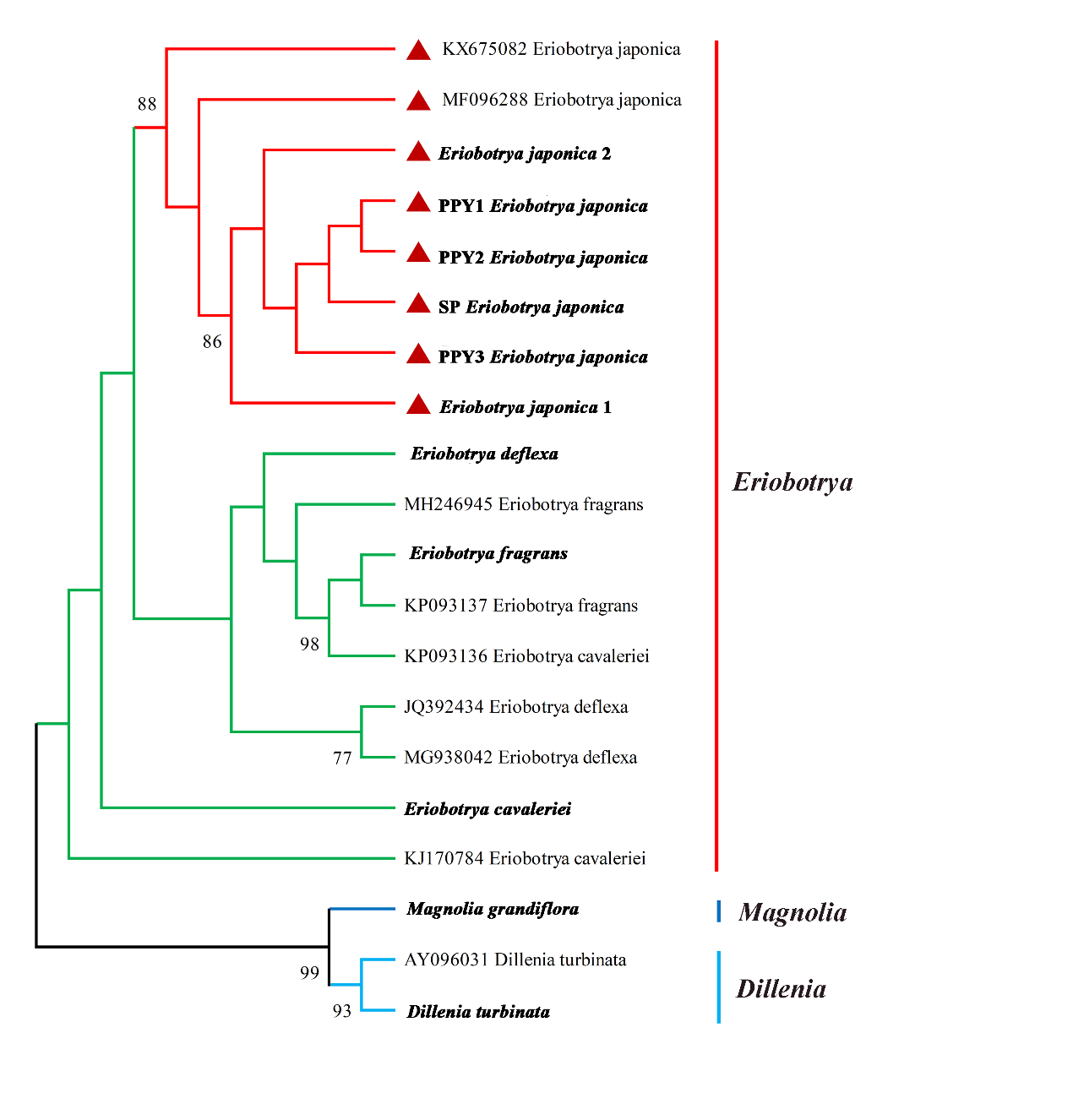


**Supplementary Fig. 1.** Phylogenetic tree constructed using ML tree based on ITS2 sequences. The number above the branches are bootstrap support values.

**Supplementary Table 1.** Interspecific (below diagonal) and intraspecific (diagonal) genetic distance of cp genome of 6 species

|  | *Eriobotrya japonica* | *E. cavaleriei* | *E. deflexa* | *E. fragrans* | *Dillenia turbinata* | *Magnolia grandiflora* |
| --- | --- | --- | --- | --- | --- | --- |
| *Eriobotrya japonica* | 0.0000 | / | / | / | / | / |
| *E. cavaleriei* | 0.0008 | NA | / | / | / | / |
| *E. deflexa* | 0.0007 | 0.0008 | 0.0000 | / | / | / |
| *E. fragrans* | 0.0009 | 0.0010 | 0.0010 | NA | / | / |
| *Dillenia turbinata* | 0.1076 | 0.1077 | 0.1076 | 0.1076 | NA | / |
| *Magnolia grandiflora* | 0.1162 | 0.1164 | 0.1163 | 0.1163 | 0.1195 | 0.0004 |

**Supplementary Table 2.** Interspecific (below diagonal) and intraspecific (diagonal) genetic distance of ITS of 6 species

|  | *Eriobotrya japonica* | *E. cavaleriei* | *E. deflexa* | *E. fragrans* | *Dillenia turbinata* | *Magnolia grandiflora* |
| --- | --- | --- | --- | --- | --- | --- |
| *Eriobotrya japonica* | 0.0005 | / | / | / | / | / |
| *E. cavaleriei* | 0.0621 | 0.0889 | / | / | / | / |
| *E. deflexa* | 0.0285 | 0.0521 | 0.0115 | / | / | / |
| *E. fragrans* | 0.0537 | 0.0777 | 0.0425 | 0.0667 | / | / |
| *Dillenia turbinata* | 0.3097 | 0.3348 | 0.3028 | 0.3185 | 0.0172 | / |
| *Magnolia grandiflora* | 0.7254 | 0.7329 | 0.7152 | 0.7204 | 0.8665 | NA |

**Supplementary Table 3.** Interspecific (below diagonal) and intraspecific (diagonal) genetic distance of ITS2 of 6 species

|  | *Eriobotrya japonica* | *E. cavaleriei* | *E. deflexa* | *E. fragrans* | *Dillenia turbinata* | *Magnolia grandiflora* |
| --- | --- | --- | --- | --- | --- | --- |
| *Eriobotrya japonica* | 0.0026 | / | / | / | / | / |
| *E. cavaleriei* | 0.0797 | 0.1046 | / | / | / | / |
| *E. deflexa* | 0.0371 | 0.0580 | 0.0120 | / | / | / |
| *E. fragrans* | 0.1050 | 0.1232 | 0.0844 | 0.14033 | / |  |
| *Dillenia turbinata* | 0.5891 | 0.5750 | 0.5743 | 0.6060 | 0.0426 | / |
| *Magnolia grandiflora* | 0.4890 | 0.5493 | 0.4942 | 0.5133 | 0.7495 | NA |

**Supplementary Table 4.** Additional ITS/ITS2 and cpDNA sequences downloaded from the GenBank to construct ML tree

|  | *Eriobotrya japonica*-1 | *E. japonica*-2 | PPY-1 | PPY-2 | PPY-3 | SP | *E. cavaleriei* | *E. deflexa* | *E. fragrans* | *Dillenia turbinata* | *Magnolia grandiflora* |
| --- | --- | --- | --- | --- | --- | --- | --- | --- | --- | --- | --- |
| cp genome | KY085905  MN577877  NC034639 | | | | | | / | MK920282 | / | MH708162 | MN990594  JN867587  NC020318 |
| ITS | MG938044  MH711704 | | | | | | KP093136  KJ170784 | MG938042  JQ392434 | MH246945  KP093137 | AY096031 | / |
| ITS2 | KX675082  MF096288 | | | | | | / | / | / | / | / |
